# Circ_0000006 and circ_0000160 regulate hsa-let-7e-5p/UBQLN4 axis in aortic dissection progression

**DOI:** 10.1371/journal.pone.0304668

**Published:** 2024-05-31

**Authors:** Yong Liu, Liang Wang, Dongyun Lei, Xiong Tan, Weitao Jin, Ming Hou, Kai Hu, Yu Yan, Hao Wang, Chaohu Xiang, Yinglong Lai

**Affiliations:** 1 Department of Cardiovascular Surgery, Affiliated Hospital of North Sichuan Medical College, Nanchong, Sichuan, China; 2 Department of Dermatology, Tianjin Academy of Traditional Chinese Medicine Affiliated Hospital, Tianjin, China; Army Medical University, CHINA

## Abstract

Aortic aneurysms (AA) and aorta dissection (AD) are life-threatening conditions with a rising incidence and high mortality rate. Recent research has linked non-coding RNAs to the regulation of AA and AD progression. In this study, we performed circRNA sequencing, microRNA (miRNA) sequencing, and messenger RNA (mRNA) sequencing on plasma samples from AA and AD patients to identify the key circRNA-miRNA-mRNA axis involved in the transition from AA to AD. Our results showed elevated levels of circ_0000006 and circ_0000160, along with decreased levels of hsa-let-7e-5p in AD samples compared to AA samples. Predictive analysis suggested that circ_0000006 and circ_0000160 potentially target hsa-let-7e-5p, which in turn may bind to the mRNA of Ubiquilin 4 (UBQLN4). In an AD cell model using vascular smooth muscle cells (VSMCs), silencing circ_0000006 and circ_0000160 attenuated the effects of platelet-derived growth factor (PDGF)-induced phenotypic changes, proliferation, and migration. This effect was partially reversed by inhibiting hsa-let-7e-5p. Furthermore, we found that overexpression of UBQLN4 counteracted the effects of hsa-let-7e-5p, suggesting UBQLN4 as a downstream mediator of hsa-let-7e-5p. In an animal model of AD, knockdown of circ_0000006 and circ_0000160 also showed protective effects against aortic septation. Overall, our findings indicate that the upregulation of circ_0000006 and circ_0000160 contributes to the progression from AA to AD by influencing abnormal phenotypic changes, migration, and proliferation of VSMCs. The Hsa-let-7e-5p/UBQLN4 axis may play a critical role in AD development. Targeting circ_0000006 and circ_0000160 could be a potential therapeutic strategy for preventing the progression of AD.

## Introduction

Thoracic aortic aneurysms (AA) and aorta dissection (AD) are severe conditions that impact the vascular wall, leading to significant enlargement of the thoracic aortic blood vessel and rupture of the aortic wall [1]. AD refers to the tearing of the adventitia into the wall of the aorta, while aneurysm denotes the abnormal growth of the aorta [2]. AD is a life-threatening disorder due to its predisposition to sudden aortic rupture, with a considerable number of patients not receiving immediate treatment [3]. Aneurysm development can occur due to significant changes in the anatomy of the aortic wall, ongoing inflammation, and oxidative stress [4, 5]. The progression from AA to acute AD is frequently fatal, necessitating regular monitoring or surgical removal of the aneurysm for high-risk patients [6]. Alternatively, intervention strategies like β-adrenergic blocking agents are proposed to delay abnormal aneurysm growth, although recent studies questioned their effectiveness [7]. Understanding the mechanisms underlying the progression from AA to AD is essential for developing novel intervention therapies.

Vascular remodeling has long been identified as a hallmark in the pathological transition of AA to AD [8]. Key players in controlling the contraction and synthesis of the aortic wall in vascular remodeling are vascular smooth muscle cells (VSMCs) [9]. The transition of VSMCs from a differentiated, contractile profile to a de-differentiated, synthesis profile, along with the accompanying infiltration of macrophages and monocytes, seems to facilitate the advancement of pathological vascular remodeling and the growth of aneurysms [10]. Contractile VSMCs are primarily found in the healthy vascular wall but can transform into synthetic VSMCs as a result of aortic trauma, high blood pressure, or arteriosclerosis [11, 12]. Consequently, patients with cardiovascular disorders like atherosclerosis and hypertension face an elevated risk of aneurysms and AD [13]. Following de-differentiation, VSMCs display impaired contractility and enhanced abilities in proliferation and migration, accompanied by decreased expression of contractile markers α-SMA and SM22α [14]. The phenotypic alteration of VSMCs plays a critical role in the development of aneurysms and the progression from aneurysms to AD [15, 16]. Hence, preserving the contractile VSMC phenotype is deemed a strategy to impede the advancement of aneurysms and AD [17]. The key to this approach lies in uncovering the molecular target responsible for mediating the switch in VSMC phenotype.

There is a growing body of evidence indicating the significant roles of non-coding RNAs (ncRNAs) in cardiovascular diseases, including AD [18, 19]. Circular RNAs (circRNAs) are a distinct class of non-coding RNAs characterized by covalent closed loop topologies and widespread expression in human cells [20]. In the regulation of endothelial permeability in the human aorta [21, 22], circRNAs play a vital role. Some circRNAs, such as circMARK3, have been suggested as potential diagnostic markers for acute AD [23]. Functioning as a type of competitive endogenous RNAs (ceRNAs), circRNAs typically act to hinder the activity of miRNAs, thereby controlling the expression of downstream miRNA targets [24]. Despite evidence indicating the dysregulation of circRNA-based ceRNA network in AD progression, there is a lack of systematic exploration regarding the mechanisms leading from AA to AD. The identification of key ncRNA players in the transition from AA to AD holds promise for potential interventions.

In the present investigation, we gathered plasma samples from healthy individuals, patients with AA and patients with AD. Subsequently, we carried out the analyses of circRNA, miRNA, and mRNA profiles to gain insights into the molecular alterations linked to the progression from AA to AD. Through our exploration, we have discovered a ceRNA regulation pathway associated with circRNA during the progression of AD. Furthermore, we also extensively examined its role in governing the transformation of VMSCs, utilizing animal experimentation to authenticate its involvement in an AD model. Our findings have unveiled an innovative ceRNA module centered around circRNA, which contributes to the advancement of AD. In addition, we have identified novel targets for therapeutic intervention.

## Materials and methods

### Medical samples collection

Plasma samples were collected from 4 healthy individuals, 4 patients with AA, and 4 patients with AD at Affiliated Hospital of North Sichuan Medical College from 10 April 2022 to 8 June 2022. The Medical Ethics Committee of Affiliated Hospital of North Sichuan Medical College provided approval for this study, and all participants signed informed consent. Diagnosis of AA and AD patients followed an established guideline [25]. Subsequently, the plasma samples were rapidly frozen in liquid nitrogen and stored at -80°C in a deep freezer until further analysis.

### Global analyses of circRNA, miRNA, and mRNA

For the profiling of circRNA, the Total RNA sample was extracted in accordance with the protocol provided by the manufacturer (Millipore-Sigma, Inc.) using TRI Reagent. Once quantified using the NanoDrop ND-1000 (Thermo Fisher Scientific, Inc.), the circRNAs were enriched by eliminating linear RNAs through the utilization of Rnase R digestion (Epicentre Biotechnologies, Inc.). Subsequently, an amplification and transcription process was employed, converting the enriched samples into fluorescent circRNA. This was achieved utilizing a random priming method kit (Arraystar Super RNA labeling kit; Arraystar, Inc.). Then, the fragmented labeled circRNA sample (1 μg) was obtained by subjecting it to a 30 min incubation at 60°C with 1 μl of 25X fragmentation buffer. Following this, the labeled circRNA was diluted with 25 μl of 2X hybridization buffer, and 50 μl of hybridization solution was dispensed on the gasket slide, which was then assembled with the circRNA expression microarray slide. The fragmented circRNAs were hybridized onto the Arraystar Human circRNA Array V2 chip (8×15K; Arraystar, Inc.). After washing the hybridized chip, signal detection was conducted using an Agilent Scanner G2505C (Agilent Technologies, Inc.).

miRNA samples were isolated from the plasma samples using the mirVana miRNA isolation kit (Ambion, Inc.) as per the supplier’s guidelines. To determine the concentration, the NanoDrop ND-1000 (Thermo Fisher Scientific, Inc.) was employed, while the RNA integrity was assessed using the Agilent Bioanalyzer 2100 (Agilent Technologies, Inc.). The Agilent Human miRNA array chip (8 x 60k; ID: 070156) was utilized for the analysis of the miRNA expression profile. The miRNA underwent amplification, dephosphorylation, denaturation, and labeling using cyanine-3-CTP (Cy3). Following purification and washing steps, the labeled RNA was hybridized onto the microarray and scanned using the Agilent Scanner G2505C (Agilent Technologies, Inc.).

mRNA: For RNA-seq analysis, the total RNA sample was extracted using TRI Reagent in accordance with the protocol provided by the manufacturer, Millipore-Sigma, Inc. The extracted samples were then re-evaluated with Qubit and Bioanalyzer, ensuring that they had a concentration of 200 ng/ml and an optical density ratio of 260/280 at 2.0. To construct the mRNA libraries, we followed the manufacturer’s protocol using the TruSeq Stranded mRNA Library Prep Kit from Illumina, Inc. The deep sequencing was carried out using the Illumina Hiseq 2500 platform. The high-throughput analysis data of this study was deposited in Sequence Read Archive database, with the accession number: PRJNA1010728.

### Differential analysis of gene expression

To analyze the circRNA microarray, the array images were examined using feature extraction software (v11.0.1.1; Agilent Technology, Inc.). For quantile normalization and subsequent data processing, the limma package from the R software package (https://www.r-project.org/) was utilized. CircRNA that exhibited a fold change (FC) ≥2 and a P<0.05 were identified as significantly differentially expressed circRNAs (DEcircRNA) between the two samples. In the case of miRNA quantification, the raw data underwent normalization using Genespring GX 12.5 software (Agilent Technologies, Inc.). A threshold of fold change ≥2.0 and a P<0.05 were employed to determine the differentially expressed miRNAs (DEmiRNA). For RNA-seq analysis, the raw reads were aligned with the Homo Sapiens GRCh38—hg38 genome version using tophat2 v2.1.0. The overall alignment rates were approximately 99%. The Cufflinks v2.2.1 software calculated the expression (FPKM) values from the alignments using the same genome/annotation. Prior to conducting differential gene expression analysis, the transcripts obtained were merged using the Cuffmerge package. The differential expression was evaluated using the Cuffdiff package, with the FDR-adjusted p value (after Benjamini-Hochberg correction for multiple testing) serving as the statistical criterion. Genes that demonstrated FDR-adjusted p < 0.05 and a fold change (FC) ≥2 were deemed to be differentially expressed (DEmRNA).

### Detection of circRNA-miRNA-mRNA interactions

To identify interactions between circRNA-miRNA and mRNA, we utilized circBase (http://www.circbase.org), a comprehensive database consolidating circRNA datasets [26]. For consistent circRNA identification, we employed CircAtlas (http://circatlas.biols.ac.cn/), an integrated resource comprising one million highly accurate circular RNAs [27]. The DEcircRNAs were then assessed for potential circRNA-miRNA interactions using CircAtlas. To predict mRNA-miRNA interactions for DEmiRNA, we employed the predictive target module of miRWalk3.0 (http://mirwalk.umm.uni-heidelberg.de/), which encompasses three distinct databases: miRDB, miRTarBase, and TargetScan. Target genes of DEmiRNAs were defined as mRNAs predicted in at least one of these databases. The final list of DEmiRNA-mRNA interactions was obtained by identifying the intersection of DEmiRNA predicted target genes and DEmRNAs identified through RNA-seq. The online Venn diagram tool (Evenn, http://www.ehbio.com/test/venn/#/) was employed for this purpose. To validate the potential DEcircRNA-DEmiRNA-DEmRNA interaction networks, we focused on shared miRNAs predicted as downstream targets of DEcircRNAs and upstream regulators of DEmRNAs. Additionally, we considered the direction of expression change to filter the interaction pairs. Finally, the Cytoscape software was utilized (Version 3.7.2, https://cytoscape.org/) to construct the final list of DEcircRNA-DEmiRNA-DEmRNA interaction axis.

### Culture of primary human VSMCs

Primary Human Aortic Smooth Muscle Cells (referred to as Human VSMCs) were procured from the ATCC, (Manassas, Virginia, United States). The cells were cultivated in human SMC medium supplied by Sciencell (San Diego, CA, USA). The cultivation took place in a humidified incubator set at 37°C with 5% CO_2_. The cells used for experimental purposes were within passages 3–10. In experiments involving PDGF treatment, the cells were exposed to PDGF-BB at a concentration of 20 ng/mL (PeproTech, NJ, USA). This treatment was conducted as specified in the experiments.

### Transfection

Cell transfection was conducted by Lipofectamine 3000 (Invitrogen, Carlsbad, CA, USA) conforming to the manufacturer’s instructions. In order to reduce circ_0000006 and circ_0000160 expressions, RiboBio Ltd. (Guangzhou, China) provided siRNAs specifically targeting these circRNAs (KD-NC). GenePharma Ltd. (Shanghai, China) prepared the pcDNA3.1 and pcDNA3.1-UBQLN4 expression vectors. To overexpress or inhibit hsa-let-7e-5p, ZSGentech Ltd. (Tianjin, China) synthesized the hsa-let-7e-5p mimic and inhibitor, respectively (inhibitor-NC, miR-NC). Transfection into VSMCs with 70% confluence in a 6-well plate involved the use of 4 μg plasmid or 200 nM siRNA, miRNA mimic, or inhibitor. After 48 hours post-transfection, the cells were collected for subsequent experiments.

### qRT-PCR

The qRT-PCR method was employed to analyze the RNA samples obtained from both cells and clinical specimens. The Beyozol total Nucleic acid extraction reagent (manufactured by Beyotime in Nanjing, China) was utilized for isolating the RNA. To assess the integrity and concentration of the RNA sample, a NanoDrop ND-1000 instrument (produced by Thermo Fisher Scientific, Inc.) was utilized.For mRNA analysis, the process involved reverse-transcription of 1 μg of total RNA into cDNA, which was carried out using the PrimeScript^™^ RT Reagent Kit (provided by Takara Biotechnology in Otsu, Japan). To analyze circRNA, the Circular RNA Synthesis Kit (manufactured by Beyotime in Beijing, China) was used. In the case of miRNA analysis, the synthesis of cDNA from total RNA was carried out utilizing the Taqman^™^ microRNA reverse transcription kit (produced by Thermo Fisher Scientific in CA, USA).The expression levels were quantified using the SYBR premix EX TAQ II kit (manufactured by Takara Biotechnology in Otsu, Japan) on the QuantStudio 3 Real-Time PCR System. The PCR cycling conditions included 40 cycles consisting of denaturation (95°C, 15 s), annealing, and extension (60°C, 30 s). Finally, the 2^–ΔΔCt^ method was employed to analyze the relative expression levels, while either GAPDH or U6 snRNA was utilized as the internal reference gene. Primer sequences used:

Human GAPDH: F’GATTCCACCCATGGCAAATTC; R’:CTGGAAGATGGTGATGGGATT
Mouse GAPDH: F’GTGGCAAAGTGGAGATTGTTG; R’:CGTTGAATTTGCCGTGAGTG
Human U6 snRNA: F’TGCGGGTGCTCGCTTCGGCAGC; R’:CCAGTGCAGGGTCCGAGGT
Mouse U6 snRNA: F’CTCGCTTCGGCAGCACA; R’:AACGCTTCACGAATTTGCGT
Human UBQLN4: F’ATTCGGGTCACCGTCAAGAC; R’:GCCTTAAACCTCCGGGAGATTT
Mouse UBQLN4: F’AGATTCGGGTCACCGTCAAGA; R’:TGAGCCTTAAACCTCCGGGA
circ_0000006: F’GACGACGACCTGAAGGAGAC; R’:GCACAAAGTAAGACGAGGAGTT
circ_0000160: F’GAGACACTCCCTACAGTTGA; R’:GCTATCGGAATAGTACCAGA
hsa-let-7e-5p: F’AGCAAGCTTTGGCACCCACCCGTAGAAC;R’:TAAGGATCCGATGCAGGGACAAGGACAGAA

### Dual luciferase assay

To determine the associations of anticipated targets, the predicted sites of WT interaction or the ones with disrupted interaction sequences (MUT) were synthesized and inserted into the PmirGLO reporter vector for luciferase activity assay (Promega, WI, USA). The plasmid reporter and the control plasmid for Renilla luciferase (hRlucneo) were co-transfected into VSMCs along with either the mimic for miR-RNA or the miR-NC in a 12-well plate, wherein the cells reached 60% confluence. Lipofectamine 3000 reagent was utilized for transfection, following the guidelines provided by the manufacturer. After 48 hours of transfection, the levels of relative luciferase activities were quantified employing the Dual-Luciferase Reporter Assay Kit (Promega, WI, USA) on a microplate reader for luminescence (Infinite 200 PRO; Tecan). The relative activity of firefly luciferase present in the plasmid reporter was standardized to the activity of the control vector for Renilla luciferase (hRlucneo).

### Cell growth analysis

CCK-8 assay was conducted to evaluate cellular growth capacity. VSMCs were transfected with either siRNA or its corresponding control. Subsequently, the cells were seeded in a 96-well plate at a density of 2000 cells per well. They were then incubated in a humidified incubator for 0, 24, 48, and 72 hours with or without PDGF treatment. At each specific time point, a volume of 10 μL CCK8 reaction solution (provided by Solarbio, Beijing, China) was added to each well. Following a 4-hour incubation period at 37°C, the culture medium was discarded. The cells present in each well were dissolved in 150 μL of DMSO for 15 minutes. The optical density (OD value) of each well was measured at a wavelength of 450nm using a Synergy H1 microplate reader (manufactured in Winooski, Vermont, USA).

### Cell migration experiment

Scratch assay was employed to assess cell migration capacity. Cell images were taken using an inverted light microscope (Leica AM6000 microscope) at 0 hour and 48 hours after incubation of VSMCs in a 6-well plate at approximately 80% confluence. A scratch was created in the middle region of each well at 0 hour time point, and the cells were then incubated at 37°C for a duration of 48 hours.

### Western blot analysis

VSMCs were lysed using RIPA buffer on ice for a duration of 15 minutes. The BCA protein assay kit (Zeye Biotech, Shanghai, China) was used to determine the protein concentration in the lysate. To perform the separation of protein samples, 10 μg of protein was loaded onto a 10% SDS-PAGE gel. Subsequently, the proteins were transferred onto a polymer film for a time period of 100 minutes at a constant current of 300 mA. Following the transfer, the membrane was incubated with specific antibodies against different proteins: α-SMA (1:1000, ab210557, Abcam), SM22α (1:1000, ab14106, Abcam), MYH11 (1:1000, ab224804, Abcam), beta-actin (1:2000, ab216070, Abcam), and UBQLN4 (1:1000, ab106443, Abcam). To detect the antibody binding, an HRP-conjugated Goat Anti-Rabbit IgG (H+L) secondary antibody (1:3000; Cell signaling Technologies, MA, USA) was applied to the membrane for 60 minutes at room temperature. After a washing step, the membrane was treated with ECL Western blot reagent (Biovision, Beijing, China) for a duration of 3 minutes, and the resulting image was captured using the GelDoc imager system (Bio-Rad, CA, United States). To analyze the density of the protein bands, Image J software (Bethesda, MD, USA) was utilized.

### Experimental animal model of AD

Four-week-old BALB/c mice were obtained from Beijing HFK Bioscience Co., Ltd. (Beijing, China). The mice were divided into 5 groups (n = 6 mice per group) as follows: 1. Control group: mice were fed a regular chow diet and provided with drinking water. 2. Model group: mice were fed a regular diet and given drinking water containing 0.1 g/kg/day of 3-aminopropionitrile fumarate salt (BAPN) for 28 days. Afterward, they were subjected to subcutaneous injection of angiotensin II for 16 days, with the dosage adjusted over time (1.5 mg/kg/day on days 1–7, 0.75 mg/kg/day on days 8–14, and 0.375 mg/kg/day on days 15–16). 3. Model+sh-NC group: mice in the model group received an administration of 0.2 mL Adeno-associated virus (AAV) carrying scramble shRNA on day 22 of week 4. 4. Model+sh-circ_0000006 group: mice in the model group received an administration of 0.2 mL AAV carrying circ_0000006 shRNA on day 22 of week 4. 5. Model+sh-circ_0000160 group: mice in the model group received an administration of 0.2 mL AAV carrying circ_0000160 shRNA on day 22 of week 4. The AAV preparation, at a concentration of 1000 pfu/mL, was provided by Genechem Ltd. (Shanghai, China), and was injected around the thoracic aorta arch three times on day 22 before the induction of AD by administration of angiotensin II. A contrast dye is then injected intravenously, which travels through the bloodstream and outlines the aorta and the branches based on X-ray images. The administration of AAV was performed around the thoracic aorta arch based on the location of the aorta. At the end of the model construction, mice were euthanized by cervical dislocation after being anesthetized with 3% sodium pentobarbital at a dose of 30mg/kg by intraperitoneal injection. The thoracic aortas were collected and subjected to histological analysis. The animal protocol received approval from Medical Ethics Committee of Affiliated Hospital of North Sichuan Medical College (No. 2022ER136-1).

### Histological analysis of aortic tissues

To ensure the integrity of the scientific analysis, aorta samples underwent proper histological staining procedures. Firstly, the aorta samples were subjected to fixation in a 4% solution of PFA for a duration of 24 hours. Subsequently, the fixed samples were embedded in paraffin and skillfully sectioned into 5-μm slices. H&E staining was executed utilizing the H&E Stain Kit (ab245880, Abcam) according to standardized protocols.To commence the staining process, the deparaffinized and re-hydrated sections were meticulously incubated in appropriate Hematoxylin, Mayer’s (Lillie’s Modification) solution for a period of 5 minutes. Following this, the sections were rinsed twice with distilled water and incubated with Bluing Reagent for precisely 2 minutes. Once the distilled water wash was completed, the sections were subjected to dehydration using absolute alcohol. Subsequently, the sections were stained using Eosin Y Solution, ensuring complete tissue coverage for a duration of 5 minutes. Afterward the section was then rinsed thrice with absolute ethanol before being mounted on a slide. Finally, under the observation of a light microscope, high-quality images were captured at 200 x magnification.

### Statistics

In this study, the statistical analysis was carried out using the SPSS 20.0 software (IBM SPSS, NY, USA). Each quantitative experiment was replicated thrice, and all data were reported as mean±SD. To determine the statistical difference between two groups, an unpaired student’s t-test was performed, while comparisons of multiple groups were analyzed using one-way analysis of variance (ANOVA) followed by Tukey’s post hoc test. For the evaluation of data at different time points in various groups, a two-way ANOVA was applied. A significance level of P < 0.05 was adopted to determine statistical significance.

## Results

### Identification of circ_0000006 and circ_0000160 /hsa-let-7e-5p/UBQLN4 ceRNA network in AA to AD progression

**Fig 1:**
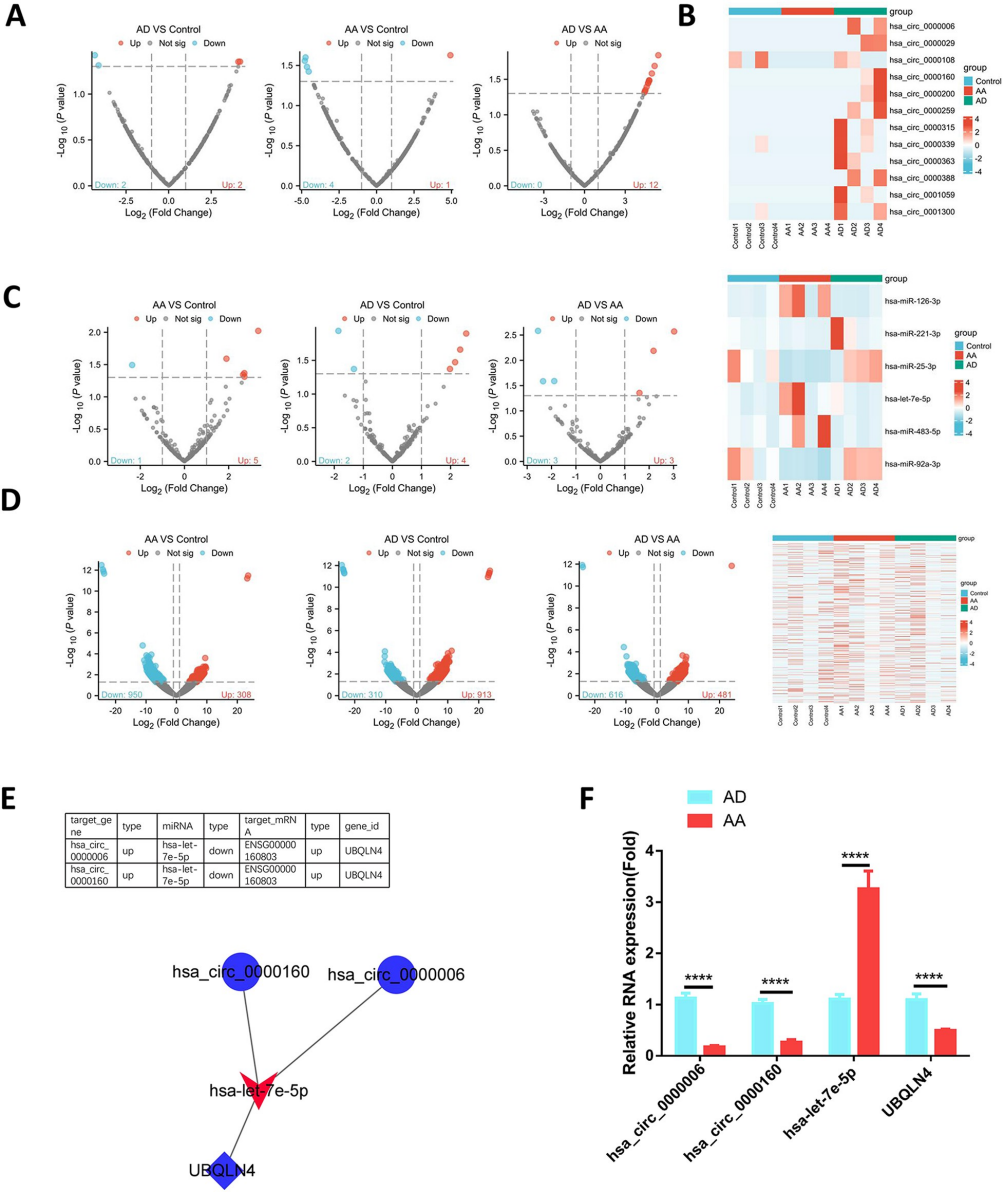
Detection of circ_0000006 and circ_0000160/hsa-let-7e-5p/UBQLN4 ceRNA network during the progression from AA to AD. (A) Volcano plots illustrating the differential expression of DEcircRNAs between AA and control, AD and control, and AD and AA samples. (B) Identification of up-regulated circRNAs in AD samples compared to AA samples. (C) Volcano plot depicting the miRNA expression differences between AA and AD samples, and the dysregulated DEmiRNAs between them. (D) Volcano plot representing the mRNA expression disparities between AA and AD samples, and the dysregulated DEmRNAs between them. (E) Interaction network illustrating the DEcircRNA-DEmiRNA-DEmRNA connections in both AA and AD samples. (F) qRT-PCR analysis conducted to evaluate the expression levels of circ_0000006, circ_0000160, hsa-let-7e-5p, and UBQLN4 between AA and AD samples. *p<0.05; **p<0.01; ***p<0.001; ****P<0.0001.

To investigate DEcircRNAs during the progression from AA to AD, a circRNA microarray was conducted on plasma samples obtained from healthy controls, AA patients, and AD patients. Through pairwise differential expression analysis, it was observed that among AA samples compared to controls, one circRNA displayed significant up-regulation while four circRNAs showed down-regulation. In the case of AD samples compared to controls, two circRNAs were up-regulated and two were down-regulated (Fig 1A and 1B). Notably, when comparing AD to AA samples, a total of twelve circRNAs were found to be up-regulated in the plasma (Fig 1A). The essential details regarding the differential expression of these twelve up-regulated circRNAs in AD are summarized in Fig 1B. Additionally, miRNA and mRNA profiling was also conducted between AA and AD samples. It was observed that in AD samples compared to AA, three miRNAs were up-regulated and three were down-regulated (Fig 1C). At the mRNA level, a substantial number of genes displayed alterations in expression, with 109 genes showing up-regulation and 233 genes exhibiting down-regulation in AD samples (Fig 1D).

In our study, we aimed to identify potential interactions among circRNAs, miRNAs and mRNAs that exhibit differential expressions between AA and AD samples. To achieve this, we utilized predictive algorithms to determine the miRNA targets of the differentially expressed circRNAs (DEcircRNAs) and the mRNA targets of the differentially expressed miRNAs (DEmiRNAs). By intersecting the predicted DEmiRNA targets with the DEmRNAs identified through RNA-seq, we formed a final list of DEmiRNA-mRNA interactions.To construct the potential networks of DEcircRNA-DEmiRNA-DEmRNA interactions, we considered the shared miRNAs that were predicted as downstream targets of the DEcircRNAs and the upstream regulators of the DEmRNAs. Through these analyses, we discovered an interaction network consisting of circ_0000006, circ_0000160, hsa-let-7e-5p, and UBQLN4. Specifically, circ_0000006, circ_0000160, and UBQLN4 exhibited up-regulation in AD samples, while hsa-let-7e-5p displayed down-regulation (Fig 1E). To validate our findings, we performed qRT-PCR analysis on clinical samples, confirming the expression patterns of the aforementioned DEcircRNA, DEmiRNA, and DEmRNA (Fig 1F). These results prompted us to further investigate the role of the ceRNA axis in regulating the functional phenotype of vascular smooth muscle cells (VSMCs) and its potential contribution to the progression from AA to AD.

### Suppression of PDGF-induced phenotypic switch, proliferation, and migration of VSMCs through knockdown of circ_0000006 and circ_0000160

**Fig 2:**
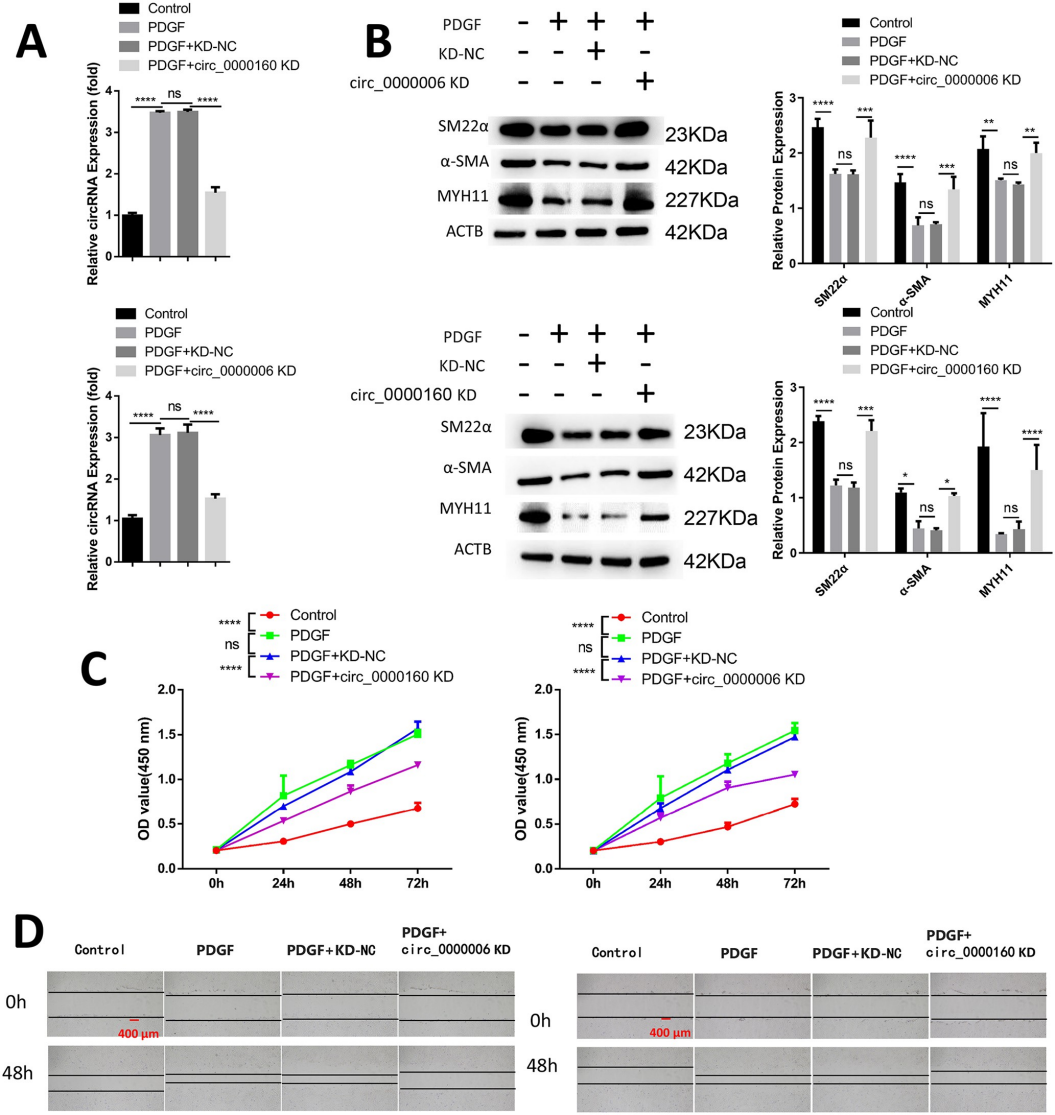
Inhibition of circ_0000006 and circ_0000160 impedes the phenotypic transition, proliferation, and migration of vascular smooth muscle cells (VSMCs) induced by platelet derived growth factor (PDGF). PDGF, at a concentration of 20 ng/mL, was utilized to induce de-differentiation of VSMCs as a cell model over a period of 48 hours. In the experimental group, VSMCs were transfected with control siRNA (KD-NC) or siRNA targeting circ_0000006 and circ_0000160 for 48 hours prior to PDGF induction. (A) Expression levels of circ_0000006 and circ_0000160 were assessed using qRT-PCR under the aforementioned conditions. (B) Western blot analysis was performed to determine the expression of contractile markers (α-SMA, SM22α, and MYH11). (C) Cell proliferation was assessed using the CCK-8 assay at 0, 24, 48, and 72 hours. (D) Wound healing assay was conducted to analyze cell migration. The presented data represents the average of three independent experiments. Significance levels were denoted as follows: *p<0.05; **p<0.01; ***p<0.001; ****P<0.0001.

In this study, we aimed to dissect the functional engagement of circ_0000006 and circ_0000160 in human vascular smooth muscle cells (VSMCs), which are crucial cells in the vascular pathophysiological responses of the aortic wall. We utilized PDGF treatment to induce the phenotypic switch in VSMCs, as it is known to play a role in VSMC de-differentiation and vascular remodeling in AA [28]. Our findings revealed that the expression of circ_0000006 and circ_0000160 was significantly elevated in PDGF-treated VSMCs. However, when siRNA was transfected, the up-regulation of circ_0000006 and circ_0000160 in PDGF-treated VSMCs was effectively suppressed (Fig 2A). To further investigate the impact of circ_0000006 and circ_0000160 on VSMCs, we analyzed the expression of contractile markers α-SMA, SM22α, and MYH11. As expected, PDGF-induced de-differentiation of VSMCs led to the down-regulation of these contractile markers. Interestingly, silencing of circ_0000006 and circ_0000160 separately promoted the expression of these contractile markers, indicating that their knockdown suppresses the PDGF-induced phenotypic switch (Fig 2B). Furthermore, the knockdown of circ_0000006 and circ_0000160 significantly inhibited PDGF-induced VSMC proliferation and migration (Fig 2C and 2D). Collectively, our results demonstrate that the up-regulation of circ_0000006 and circ_0000160 contributes to the PDGF-induced phenotypic switch, proliferation, and migration in VSMCs.

### hsa-let-7e-5p functions as a downstream mediator of circ_0000006 and circ_0000160

**Fig 3:**
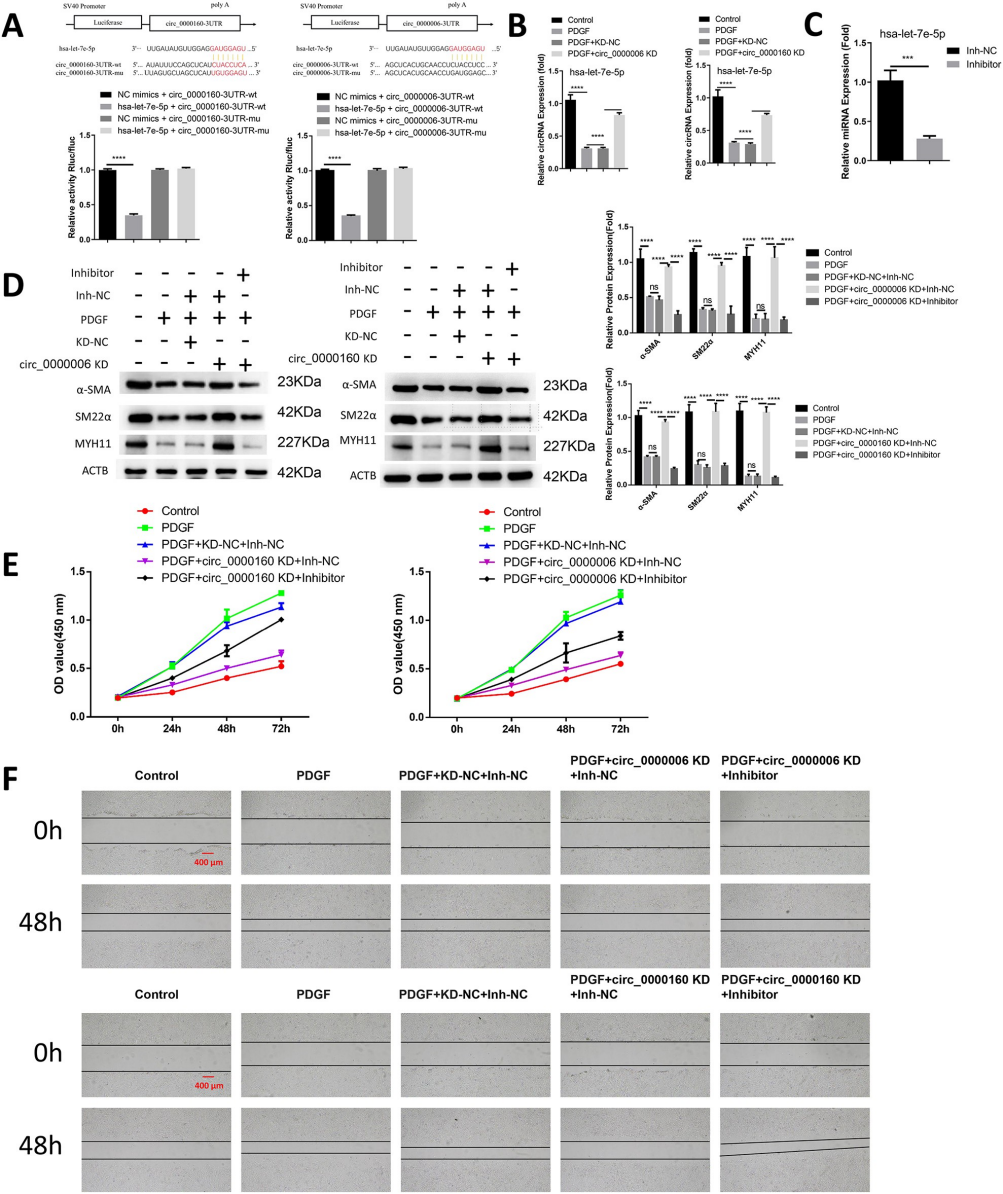
hsa-let-7e-5p acts as a downstream mediator of circ_0000006 and circ_0000160. (A) The effect of miR-NC or hsa-let-7e-5p mimic was assessed in a dual luciferase reporter analysis of WT and MUT reporter of circ_0000006 or circ_0000160. (B) Upon PDGF treatment and the silencing of circ_0000006 or circ_0000160, hsa-let-7e-5p expression in VSMCs was analyzed using qRT-PCR. (C) The transfection of inhibitor control (Inh-NC) or hsa-let-7e-5p inhibitor in VSMCs was followed by qRT-PCR analysis of hsa-let-7e-5p expression. (D-F) VSMCs were treated with PDGF for 48 hours after transfecting them with control siRNA (KD-NC), circ_0000006 or circ_0000160 siRNA, along with control inhibitor (Inh-NC) or hsa-let-7e-5p inhibitor. (D) Western blot detection was performed to assess contractile markers (α-SMA, SM22α, and MYH11). (E) Additionally, a CCK-8 proliferation assay was performed at 0, 24, 48, and 72 hours. (F) Cell migration analysis was conducted using a wound healing assay. Data represent the summary of three independent experiments. Statistical significance is denoted as *p<0.05; **p<0.01; ***p<0.001; ****P<0.0001.

After identifying hsa-let-7e-5p as the differentially expressed microRNA and the target of circ_0000006 and circ_0000160, our next objective was to validate their functional interaction. To achieve this, we conducted a dual luciferase reporter assay, using both wild type (WT) and mutated (MUT) binding sites. The results demonstrated that overexpressing hsa-let-7e-5p through mimic transfection effectively inhibited the luciferase activity of the WT reporter, while no significant inhibition was observed in the MUT reporter (Fig 3A). This finding supports the notion that their interaction occurs through the predicted wild type binding sequences. Moreover, we observed that hsa-let-7e-5p expression in VSMCs was repressed upon treatment with PDGF, but this repression was rescued when circ_0000006 and circ_0000160 were silenced (Fig 3B). Subsequently, we employed an hsa-let-7e-5p inhibitor, which successfully suppressed hsa-let-7e-5p expression (Fig 3C), to investigate whether hsa-let-7e-5p mediates the effects of circ_0000006 and circ_0000160. Performing an analysis of contractile markers (α-SMA, SM22α, and MYH11), we discovered that silencing circ_0000006 or circ_0000160 effectively suppressed PDGF-induced phenotypic switch. Moreover, this effect was abrogated when hsa-let-7e-5p was inhibited (Fig 3D). Additionally, hsa-let-7e-5p inhibition also abolished the effects of circ_0000006 and circ_0000160 knockdown on PDGF-induced cell proliferation and migration (Fig 3E and 3F). These findings collectively indicate that hsa-let-7e-5p acts as a downstream mediator of circ_0000006 and circ_0000160 in the PDGF-induced de-differentiation of VSMCs.

### hsa-let-7e-5p over-expression inhibits PDGF-stimulated phenotypic switch, proliferation and migration in VSMCs

**Fig 4:**
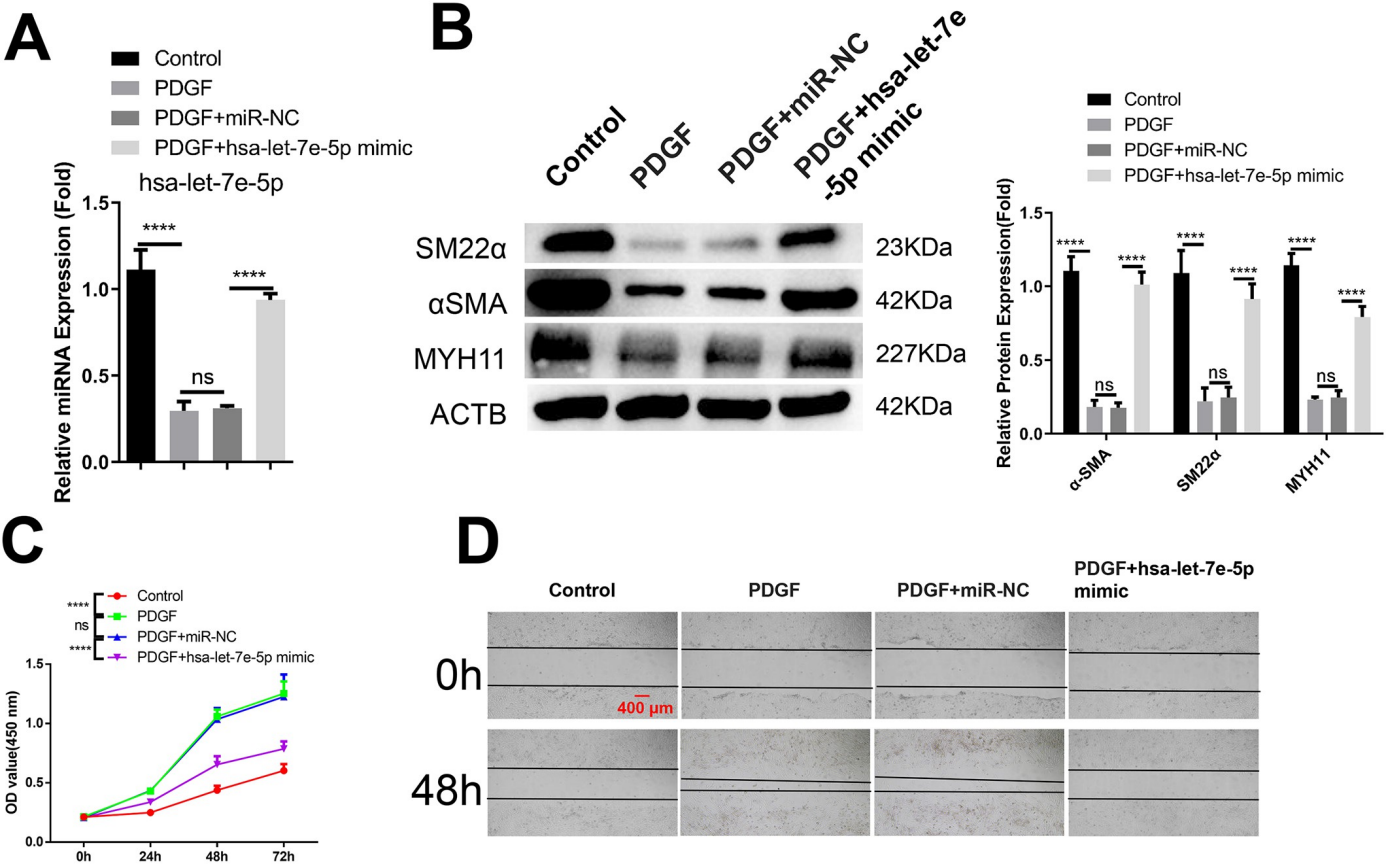
The expression of hsa-let-7e-5p restricts the phenotypic switch, proliferation, and migration induced in VSMCs. VSMCs were transfected with either a control miRNA mimic (miR-NC) or a mimic of hsa-let-7e-5p, and subsequently treated with PDGF for a duration of 48 hours. (A) The quantification of hsa-let-7e-5p expression in the aforementioned conditions through qRT-PCR analysis. (B) Western blot detection of contractile markers, including α-SMA, SM22α, and MYH11. (C) The assessment of proliferation using the CCK-8 assay, with time points measured at 0, 24, 48, and 72 hours. (D) Analysis of cell migration via the wound healing assay. The data presented represents the aggregate of three independent experiments. Statistical significance is denoted by *p<0.05; **p<0.01; ***p<0.001; ****P<0.0001.

To demonstrate the direct impact of hsa-let-7e-5p on VSMCs, cells treated with PDGF were transfected with either a miRNA control (miR-NC) or a mimic of hsa-let-7e-5p. The expression of hsa-let-7e-5p was significantly enhanced by the miRNA mimic following PDGF treatment (Fig 4A). The suppressive effects of PDGF on the expression of contractile markers were mostly reversed by the hsa-let-7e-5p mimic (Fig 4B). Consistent findings were observed in assays assessing cell proliferation and migration, where overexpression of hsa-let-7e-5p hindered PDGF-induced proliferation and migration in VSMCs (Fig 4C and 4D). Thus, it can be concluded that hsa-let-7e-5p directly regulates the de-differentiation of VSMCs induced by PDGF.

### The modulation of UBQLN4 expression by hsa-let-7e-5p controls the phenotypic switch observed in VSMCs

**Fig 5:**
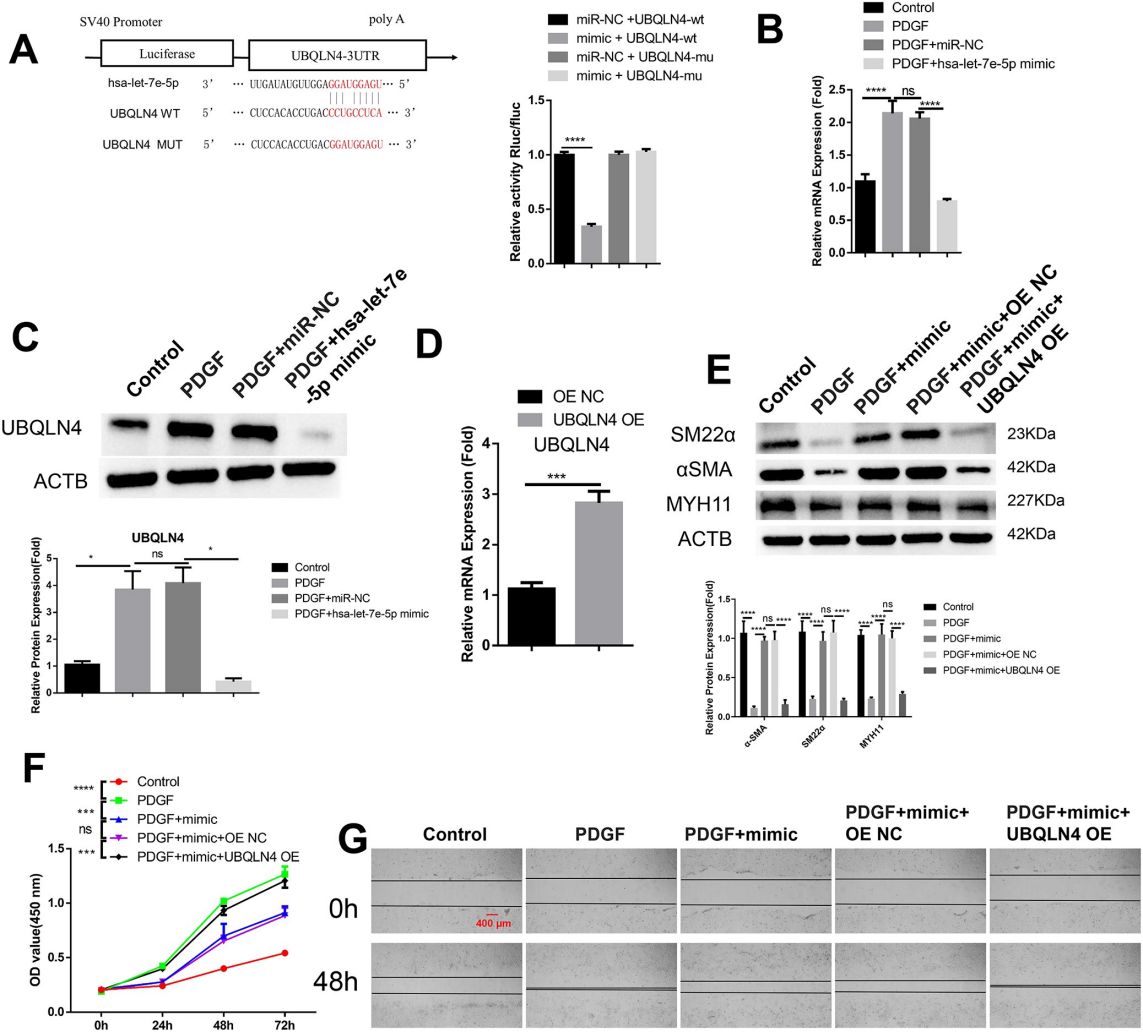
The expression of UBQLN4 is regulated by hsa-let-7e-5p to control the phenotypic switch in VSMCs. (A) The influence of miR-NC or hsa-let-7e-5p mimic on WT and MUT reporter of UBQLN4 was analyzed using dual luciferase reporter assay. (B-C) VSMCs were exposed to PDGF for 48 hours after transfection with miR-NC or hsa-let-7e-5p mimic as controls. (B) qRT-PCR was conducted to measure the mRNA level of UBQLN4, while (C) Western blot analysis was performed to assess the protein level of UBQLN4. (D) qRT-PCR analysis evaluated UBQLN4 mRNA expression in VSMCs transfected with either the empty vector (OE NC) or UBQLN4 expression vector (UBQLN4 OE). (E-G) VSMCs were transfected with hsa-let-7e-5p mimic in combination with either the empty vector (OE NC) or UBQLN4 expression vector (UBQLN4 OE), followed by treatment with PDGF for 48 hours. (E) Western blot detection was utilized to determine the levels of contractile markers (α-SMA, SM22α, and MYH11). (F) Cell proliferation was assessed using the CCK-8 assay at 0, 24, 48, and 72 hours. (G) Wound healing assay was conducted to analyze cell migration. The data presented are from three independent experiments. Statistical significance is denoted as *p<0.05; **p<0.01; ***p<0.001; ****P<0.0001.

To investigate the role of the hsa-let-7e-5p/UBQLN4 axis in PDGF-induced phenotypic changes, we aimed to validate UBQLN4 as a target of hsa-let-7e-5p. We conducted a dual luciferase reporter assay using a reporter with wild type (WT) or mutated (MUT) binding sites at the 3’ UTR of UBQLN4 mRNA. The results revealed that hsa-let-7e-5p mimic inhibited the activity of the WT reporter, while no significant inhibition was observed in the MUT reporter (Fig 5A). Furthermore, we observed that PDGF treatment up-regulated the expression of UBQLN4 at the mRNA and protein levels, which was suppressed by over-expression of hsa-let-7e-5p in VSMCs (Fig 5B and 5C). To further investigate the role of UBQLN4, we used an empty vector (OE NC) and a UBQLN4 expression vector (UBQLN4 OE) to induce increased expression of UBQLN4 in VSMCs (Fig 5D). Analysis of contractile markers revealed that hsa-let-7e-5p mimic reversed the effect of PDGF treatment on the expression of these markers. However, this effect was abrogated by UBQLN4 over-expression (Fig 5E). Additionally, UBQLN4 over-expression counteracted the effects of hsa-let-7e-5p mimic on PDGF-induced proliferation and migration (Fig 5F and 5G). Taken together, these findings suggest that UBQLN4 acts as a downstream effector of hsa-let-7e-5p and plays a role in regulating PDGF-induced de-differentiation of VSMCs.

### Silencing circ_0000006 or circ_0000160 through AAV-mediated intervention shows potential in mitigating the progression of AD in a mouse model

**Fig 6:**
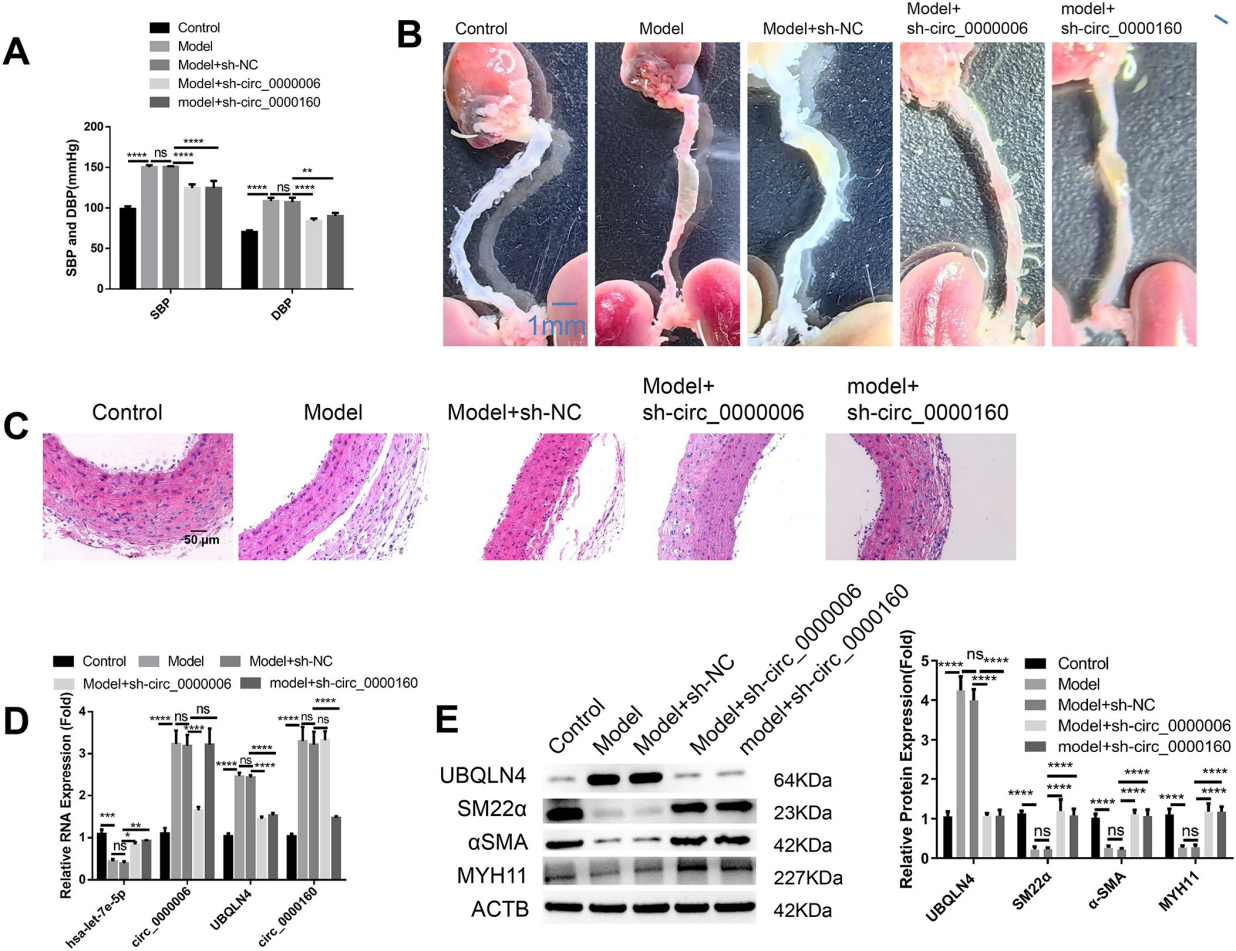
Knockdown of circ_0000006 and circ_0000160 using AAV curbs the progression of AD in a mouse model. BALB/c mice were treated with 3-aminopropionitrile fumarate salt (BAPN) for 28 days and angiotensin II for 16 days to induce AD. On Day 22, the mice in the model group were injected with Adeno-associated virus (AAV) containing scramble shRNA, circ_0000006 shRNA, or circ_0000160 shRNA. (A) Overview of body weight, systolic blood pressure (SBP), and diastolic blood pressure (DBP) in each group. (B) The aorta dissected from the model group showing the development of aortic aneurysm. (C) HE staining of the aorta sections in each group. (D) Quantitative reverse transcription-polymerase chain reaction (qRT-PCR) analysis of circ_0000006, circ_0000160, hsa-let-7e-5p, and UBQLN4 expression in the aorta wall tissues. (E) Western blot detection of contractile markers (α-SMA, SM22α, and MYH11) in the aorta wall tissues. *p<0.05; **p<0.01; ***p<0.001; ****P<0.0001.

To assess this potential, we induced the AD mouse model by administering 3-aminopropionitrile (BAPN) and injecting angiotensin II subcutaneously. In the experimental group, AAV carrying control shRNA (sh-NC), circ_0000006 shRNA, or circ_0000160 shRNA was administered respectively. Our experiment concluded with the measurement of weight, systolic blood pressure (SBP), and diastolic blood pressure (DBP) in each group. There were no significant changes in body weight among the various groups. However, both SBP and DBP showed a significant increase in the model group, while silencing of circ_0000006 shRNA or circ_0000160 partially alleviated SBP and DBP in the model group (Fig 6A). All the animals in AD and AD+sh-NC groups developed aortic aneurysm formation and clear AD abnormalities (Fig 6B and 6C). In the circ_0000006 or circ_0000160 silencing groups, there is only one animal with minor abnormality of AD, and the remaining mice exhibited improvement in aortic aneurysm formation (Fig 6B) and reduced aortic dissection in histological analysis (Fig 6C). Furthermore, qRT-PCR analysis of aortic tissues displayed up-regulation of circ_0000006, circ_0000160, and UBQLN4, as well as down-regulation of has-let-7e-5p expression in the model group compared to the control group. Silencing of circ_0000006 or circ_0000160 suppressed UBQLN4 expression and enhanced the level of has-let-7e-5p (Fig 6D). Western blot analysis of the contractile markers demonstrated down-regulation of α-SMA, SM22α, and MYH11 in the model group compared to the control, while silencing of circ_0000006 or circ_0000160 restored their expression in the model group (Fig 6E). Therefore, these findings suggest a potential role of circ_0000006 and circ_0000160 over-expression in AD progression by targeting the hsa-let-7e-5p/UBQLN4 molecular axis.

## Discussion

AA and AD are life-threatening disorders with an increasing incidence and a high mortality rate, and there is a noticeable difference in occurrence between males and females [29]. The prevalence of AD has risen significantly by 2 to 4 times in the past three decades, and the likelihood of AD development in AA patients is as high as 80% [30]. Blood-based biomarkers, such as D-Dimer or CRP, have traditionally been used for AD diagnosis. However, there have been indications of misdiagnosis and a lack of precision [31]. It is crucial to identify dependable biomarkers for early detection and intervention. In this study, we conducted an integrative analysis of circRNA, miRNA, and mRNA profiles to elucidate the transitional process from AA to AD. We discovered differential expression of circRNAs, miRNAs, and mRNAs between plasma samples of AA and AD patients. Further research is necessary to determine whether some of these molecules have the potential to function as biomarkers for AD detection.

In our investigation, we have also recognized a crucial ceRNA network that consists of Circ_0000006, circ_0000160, hsa-let-7e-5p, and UBQLN4 during the advancement from AA to AD. The functional role of this ceRNA network in the phenotypic conversion of VSMCs and its impact on an animal model of AD were further assessed in our study. Our findings suggest that circ_0000006 and circ_0000160 exhibit higher expression levels in AD samples compared to control and AA samples. Conversely, the expression level of hsa-let-7e-5p was found to be down-regulated in AD, while the expression level of UBQLN4 was up-regulated. Previous investigations have indicated the regulatory involvement of circRNAs in both AA and AD. For instance, Tian et al. [32] demonstrated dysregulation of 506 circRNAs in acute Stanford type A aortic dissection (AAAD), with circRNA3 showing promise as a diagnostic biomarker for AAAD. In a separate study, Zho et al. conducted a circRNA microarray analysis in human type A thoracic aortic dissection (TAD) tissues, observing up-regulation of 156 circRNAs and down-regulation of 106 circRNAs. They further elucidated the impact of hsa-circRNA-101238 in blocking hsa-miR-320a production in TAD, leading to increased expression of MMP9 in VSMCs [33]. Nevertheless, none of these studies specifically investigated the alterations occurring during the transition from AA to AD. Therefore, our data offers novel insights into the dysregulation of circRNAs, miRNAs, and mRNAs in the progression from AA to AD.

Vascular smooth muscle cells (VSMCs) are the primary cellular components of the aortic wall and play a crucial role in regulating blood flow and pressure [34]. An essential pathological change in aortic dissection (AD) is the disruption of the middle layer of blood vessels. α-SMA and SM22α are key protein constituents that contribute to the contraction capability of VSMCs. The down-regulation of these proteins, along with the concurrent up-regulation of matrix metalloproteinases, leads to the phenotypic transformation of VSMCs from a contractile to a synthetic phenotype [35, 36]. This shift significantly increases the risk of aortic aneurysm rupture and the development of AD. In VSMCs induced by PDGF, both circ_0000006 and circ_0000160 showed over-expression, and silencing these two circRNAs could mitigate the dedifferentiation, proliferation, and migration of PDGF-induced VSMCs. Moreover, in an animal model, the silencing of circ_0000006 and circ_0000160 through AAV-mediated intervention also suppressed AD progression. Consequently, over-expression of circ_0000006 and circ_0000160 may play a role in promoting the phenotypic transition of VSMCs during the progression from aortic aneurysm (AA) to AD.

We conducted additional verification to confirm that the downstream mediator of circ_0000006 and circ_0000160 in the regulation of the phenotypic switch of VSMCs is the hsa-let-7e-5P/UBQLN4 axis. The down-regulation of contractile markers, cell proliferation, and migration induced by PDGF can be suppressed by the hsa-let-7e-5p mimic. Conversely, the effect of the hsa-let-7e-5p mimic is counteracted by forced UBQLN4 expression. There are various instances of non-coding RNAs involved in the regulation of the VSMC phenotype. According to Ren et al. [37], LncRNA H19 regulates the smooth muscle cell phenotype by sequestering miR-193b-3p during the development of AD. Another study [38] revealed that overexpression of circTGFBR2 in VSMCs associated with AD leads to an increase in the expression of contraction markers (α-SMA and SM22α), while synthetic markers (MGP and OPN) are down-regulated. This demonstrates that circTGFBR2 suppresses the phenotypic transition of VSMCs and inhibits the progression of AD. Additionally, Zheng et al. [39] demonstrated that samples from patients with aortic rupture and aortic smooth muscle cells under hypoxic conditions exhibit abnormally high levels of circ-000595. They also identified miR19a as a target of circ-000595 in AA. However, it remains unclear whether these molecules contribute to the progression from AA to AD.

There are several unanswered questions that need to be addressed in future research. Firstly, the underlying mechanisms responsible for the over-expression of circ_0000006 and circ_0000160 during the transition from AA to AD are not well understood. It is necessary to investigate whether the dysregulation of these circRNAs is caused by hypoxia or inflammation in AA. It is important to thoroughly investigate the biological role of UBQLN4 in determining the phenotypic transition of VSMCs. Finding answers to the above-mentioned questions may provide valuable insights into new intervention strategies for the progression from AA to AD.

## Conclusions

To sum up, our study has identified a crucial ceRNA regulatory module that governs the phenotypic transition of VSMCs during the progression from AA to AD. We have observed specific up-regulation of circ_0000006 and circ_0000160 in AD samples, and their over-expression leads to the loss of contractile properties in VSMCs. These circRNAs act as upstream regulators by inhibiting the activity of hsa-let-7e-5p, resulting in the up-regulation of UBQLN4. Future research should focus on evaluating the potential of circ_0000006 and circ_0000160 as biomarkers for the progression from AA to AD, as well as investigating the mechanism by which UBQLN4 regulates the phenotypic transition of VSMCs.

## Supporting information

S1 Data (XLSX)

S1 Raw images (PDF)
