# Supplementary material for: Circ_0000006 and circ_0000160 regulate hsa-let-7e-5p/UBQLN4 axis in aortic dissection progression
Source: PLoS One. 2024 May 31;19(5):e0304668. doi: 10.1371/journal.pone.0304668 (PMC11142605; doi:10.1371/journal.pone.0304668)

Figure 2

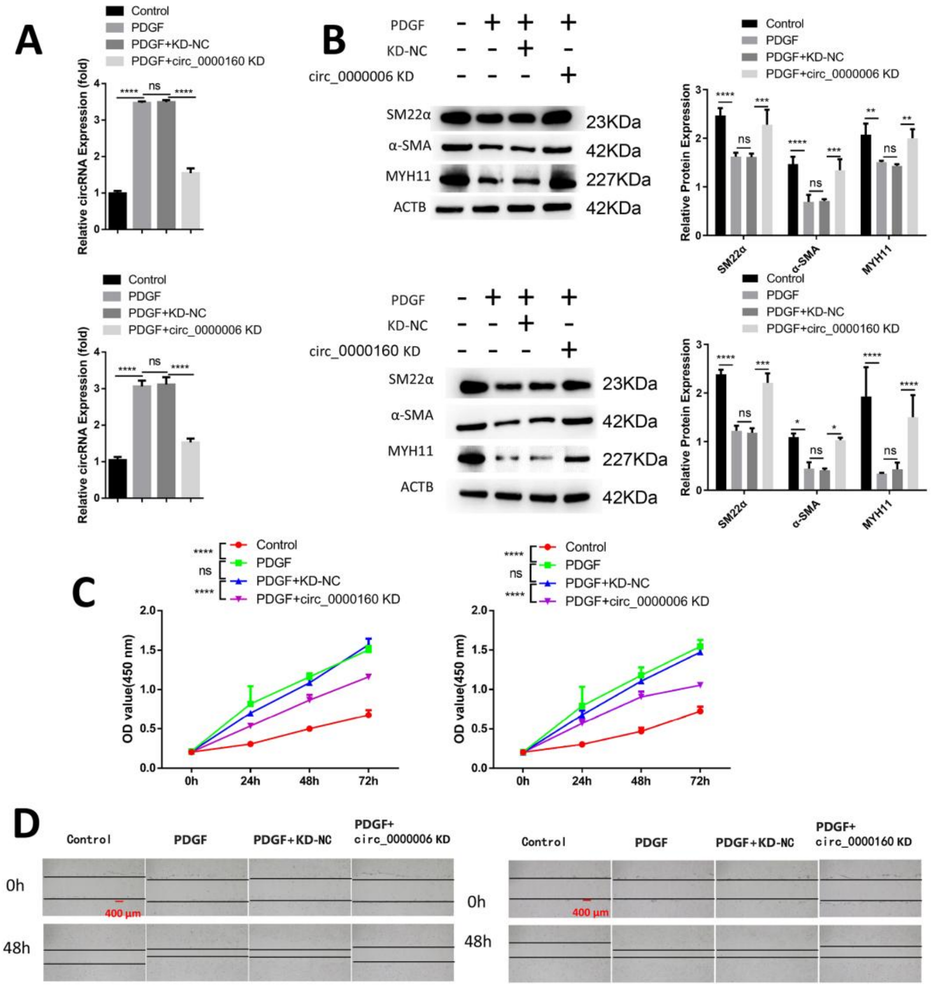

Figure 2B

Circ_circ_0000006 KD

SM22 α

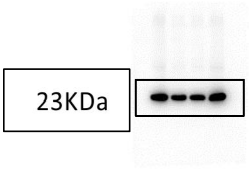

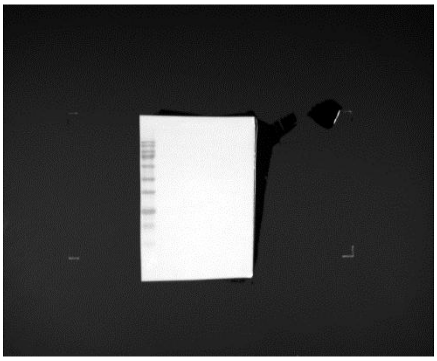

α-SMA

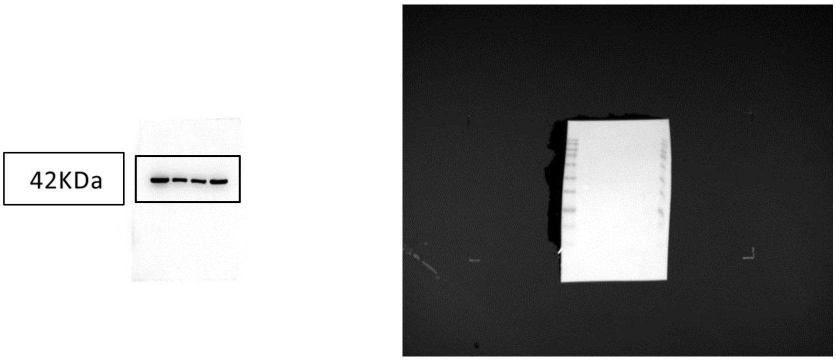

MYH11

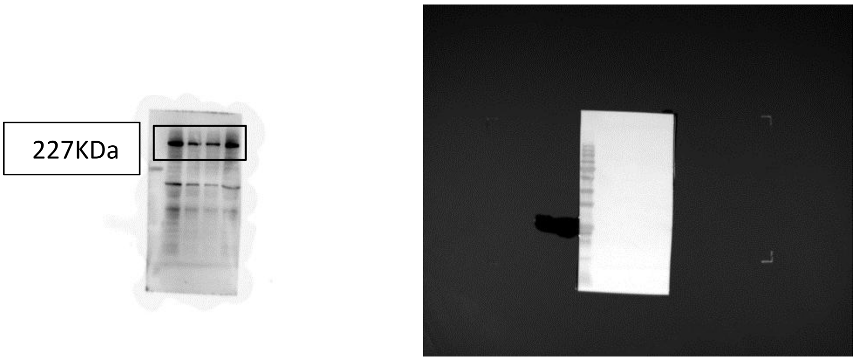

ACTB

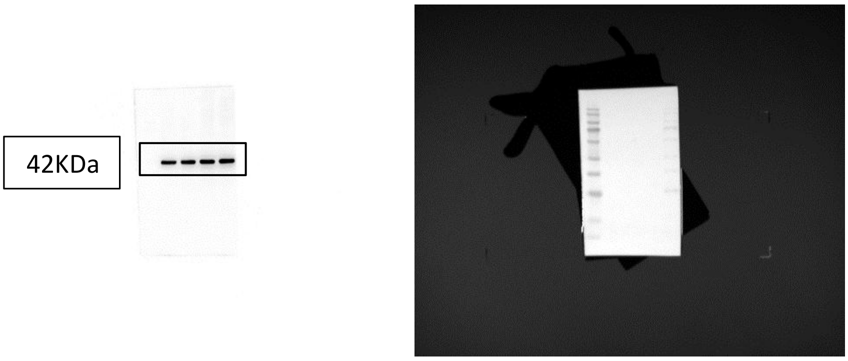

circ_0000160 KD

SM22α

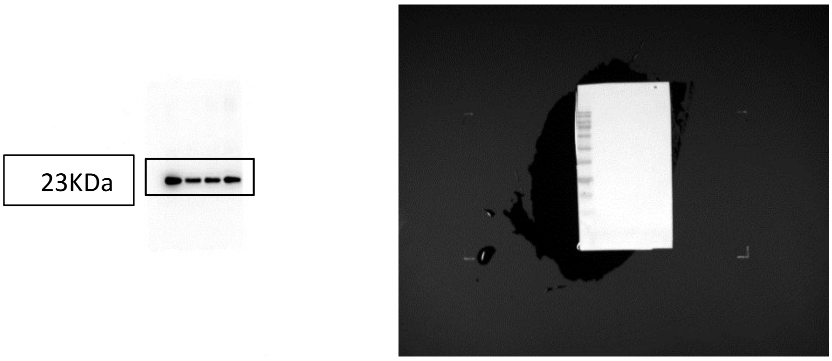

α-SMA

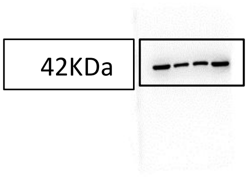

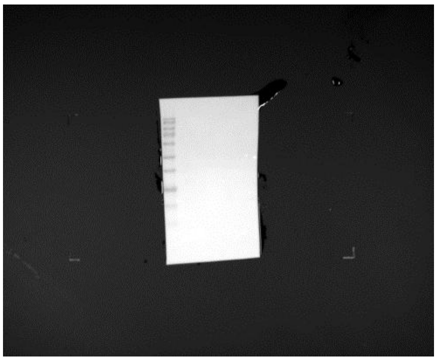

MYH11

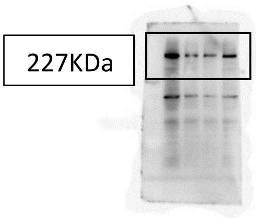

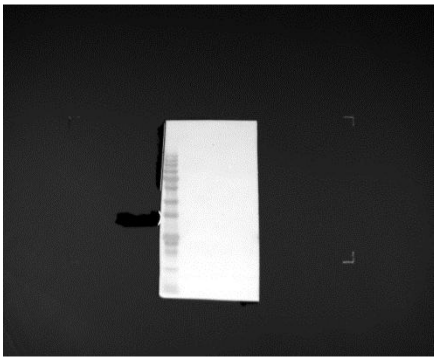

ACTB

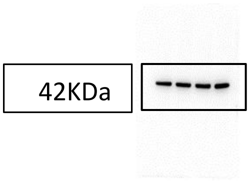

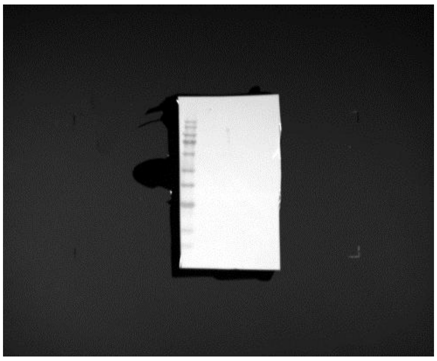

Figure 3

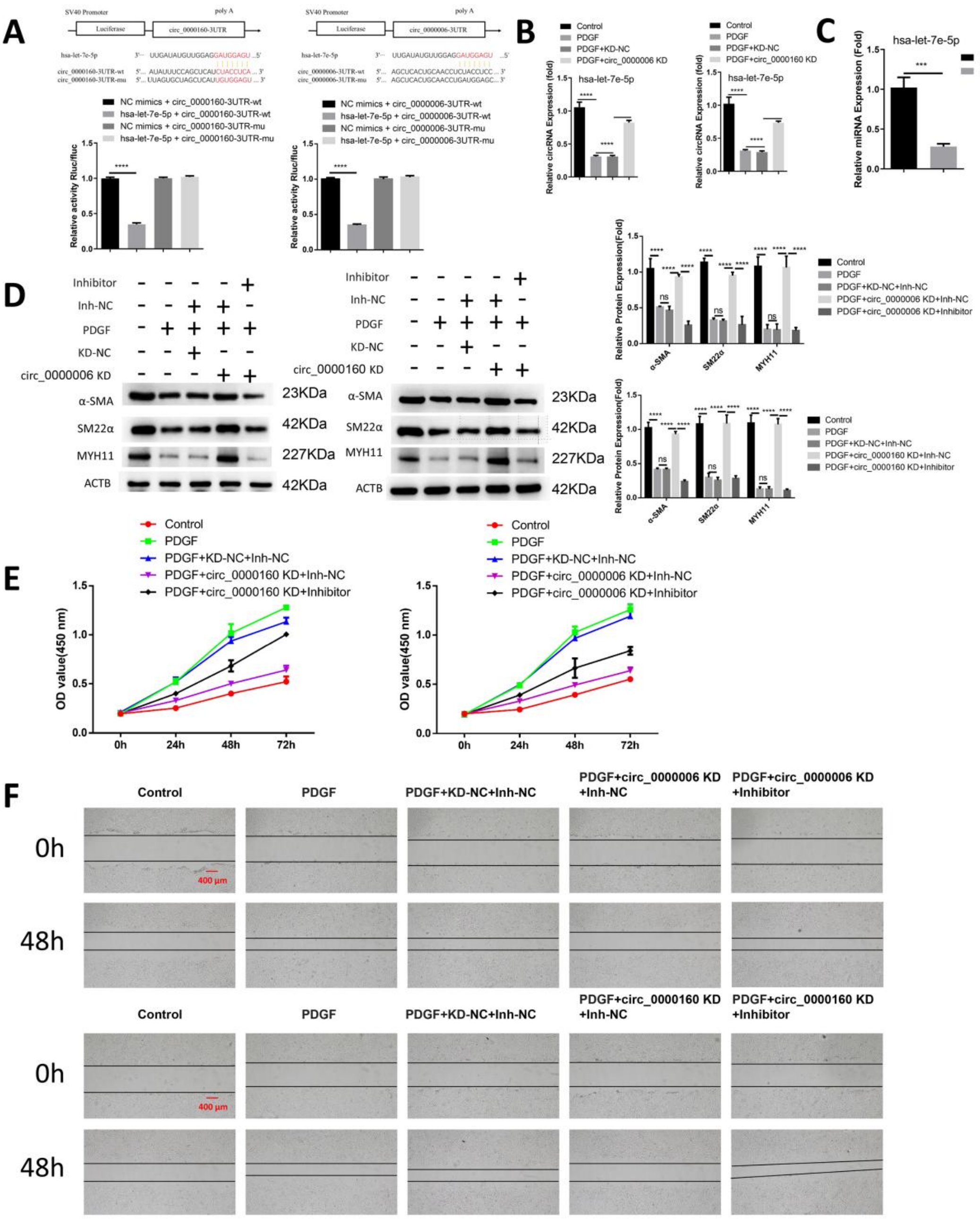

Circ_circ_0000006 KD

SM22 α

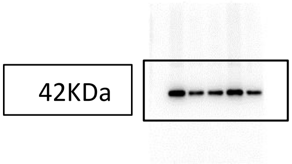

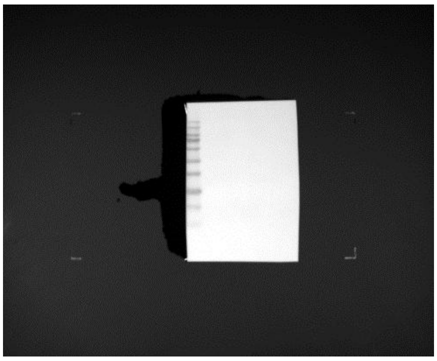

α -SMA

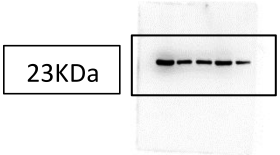

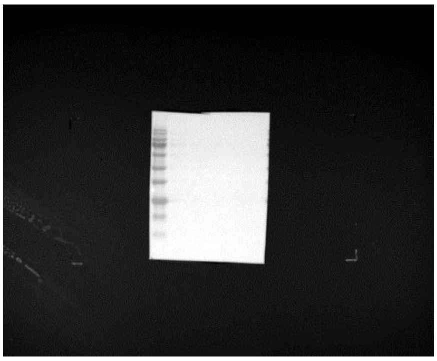

MYH11

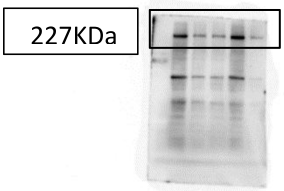

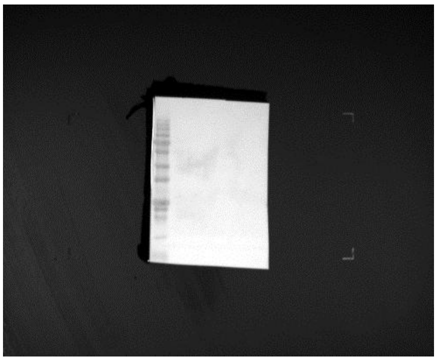

ACTB

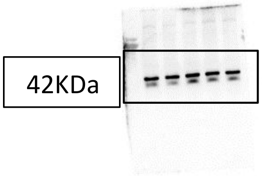

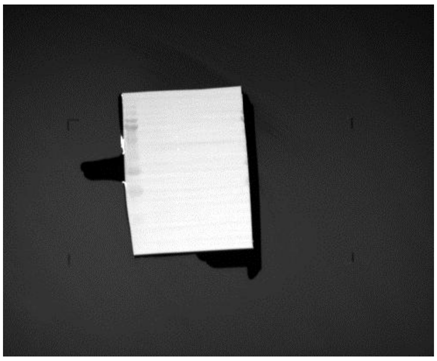

circ_0000160 KD

SM22 α

42KDa

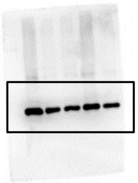

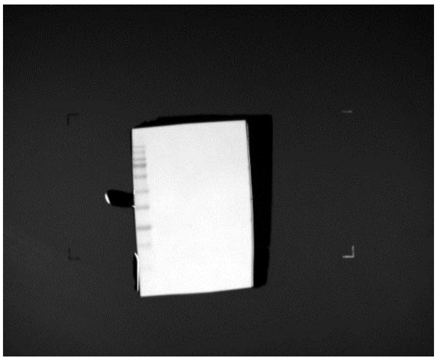

α -SMA

23KDa

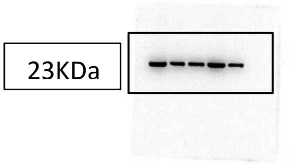

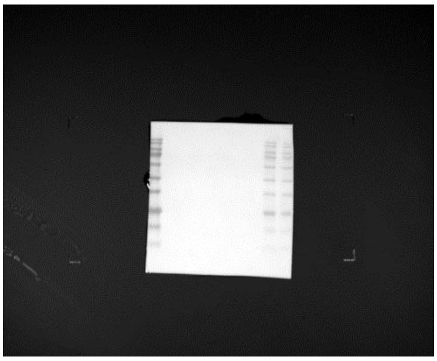

MYH11

227KDa

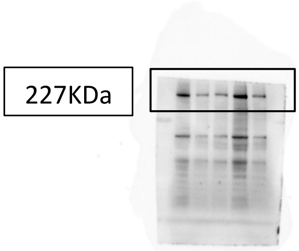

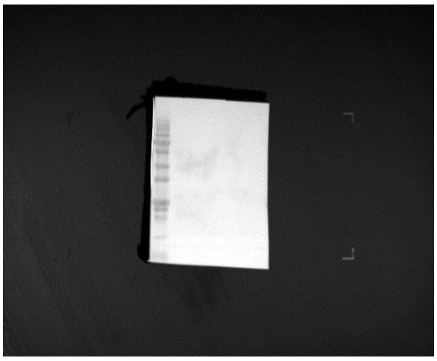

ACTB

42KDa

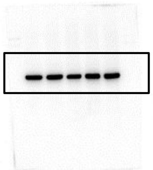

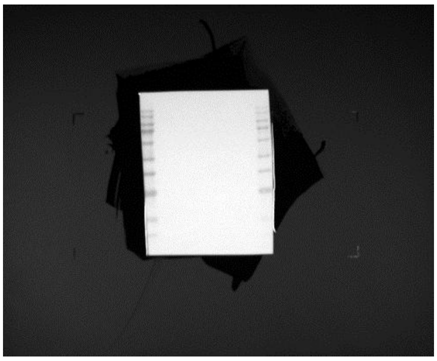

Figure 4

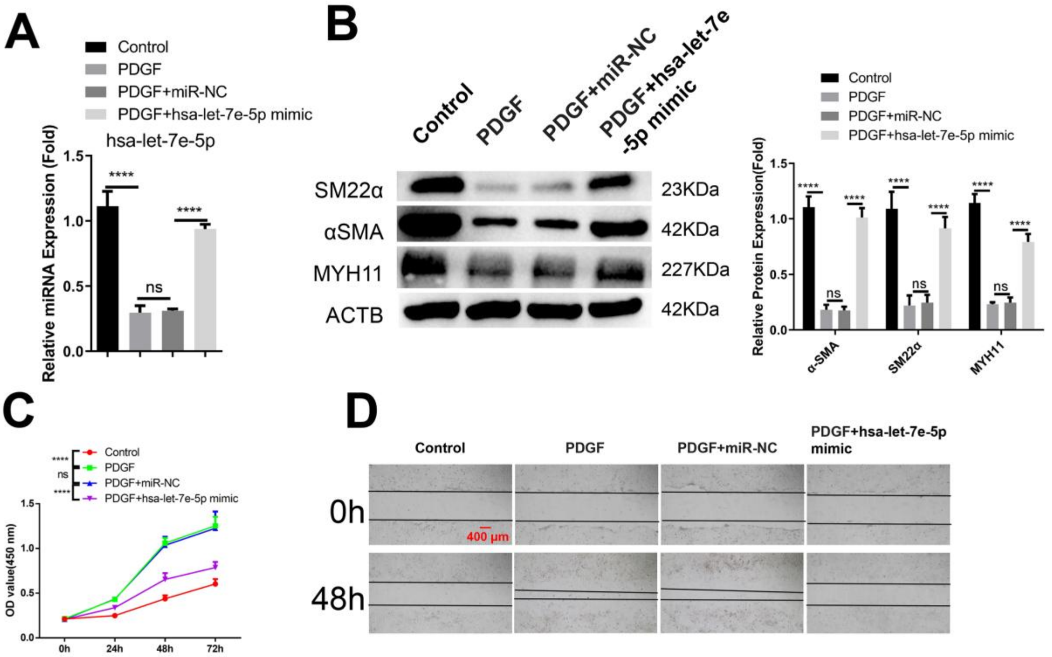

SM22 α

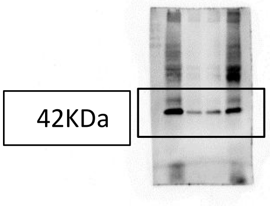

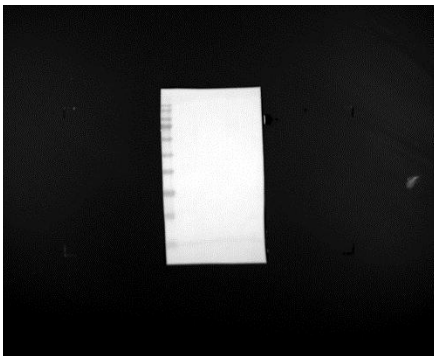

α -SMA

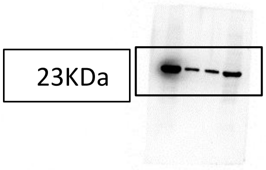

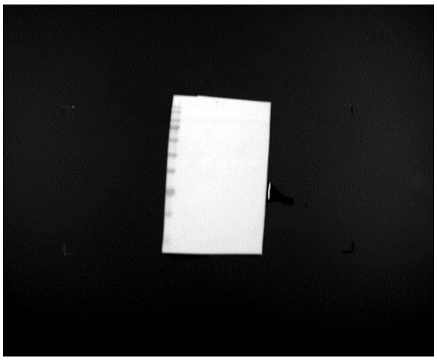

MYH11

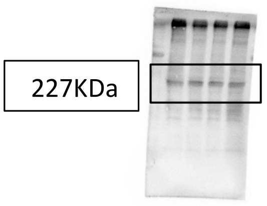

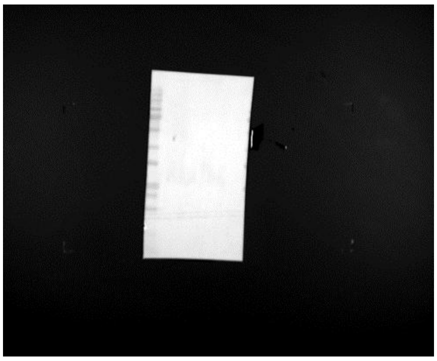

ACTB

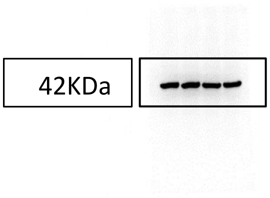

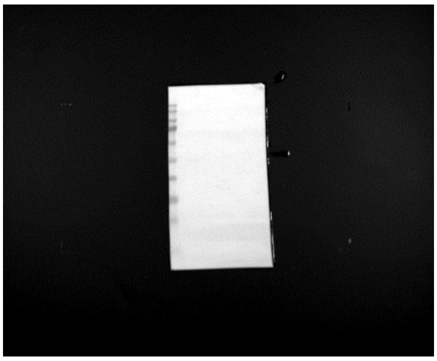

Figure 5

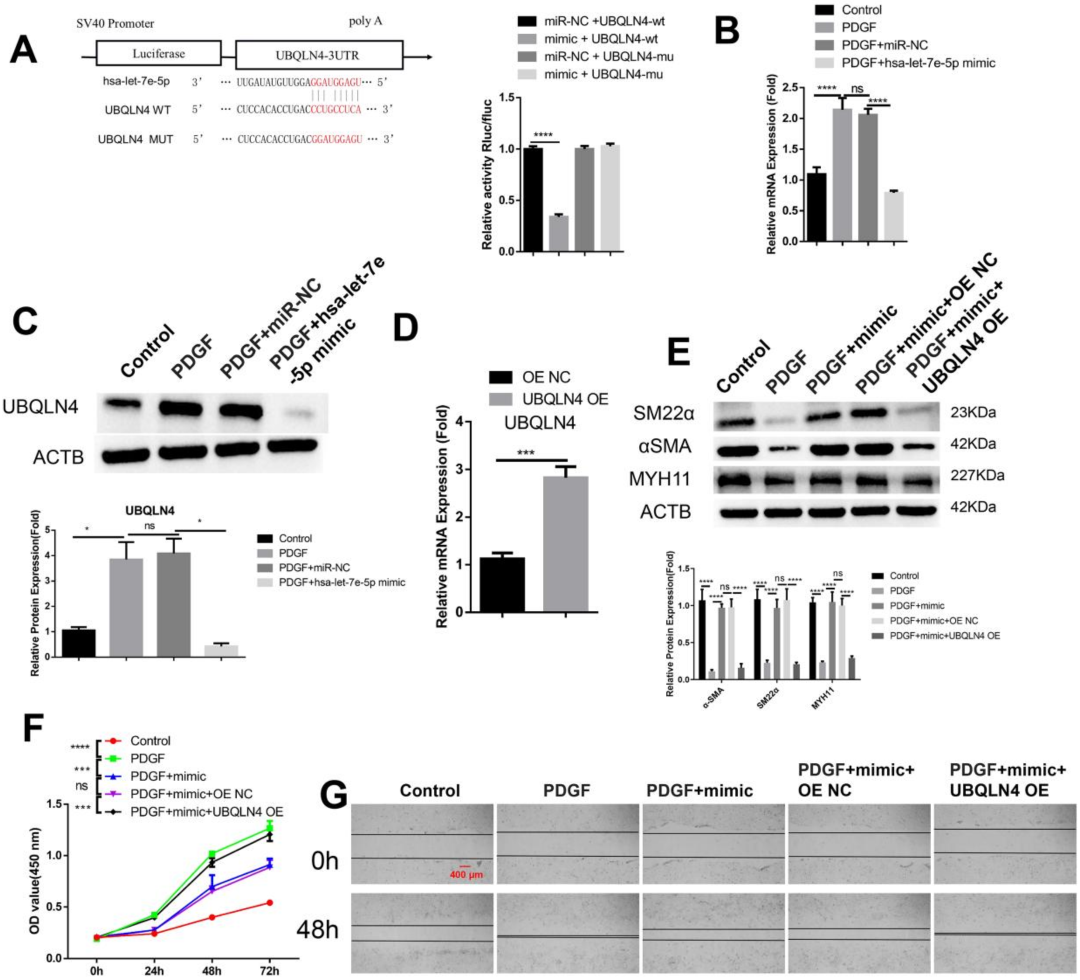

Figure 5C:

UBQLN4

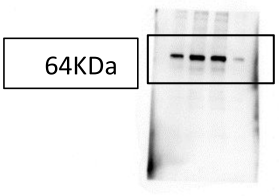

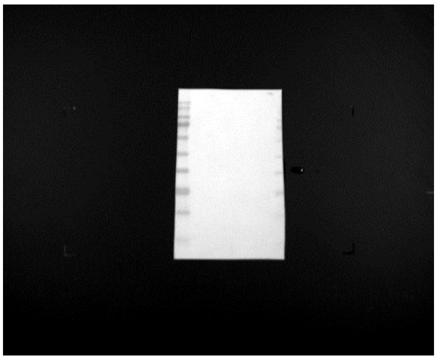

ACTB

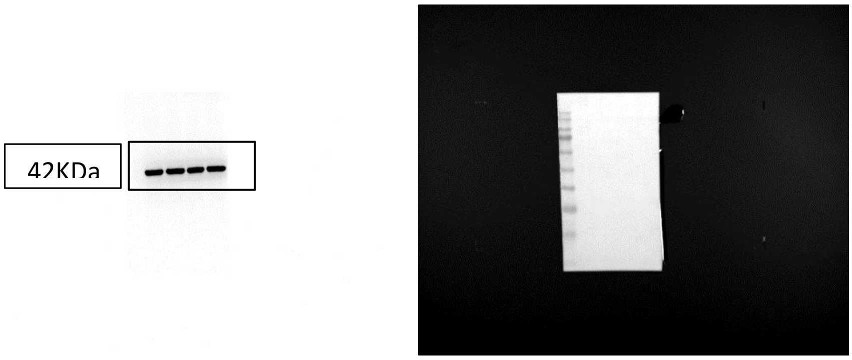

Figure 5E:

SM22α

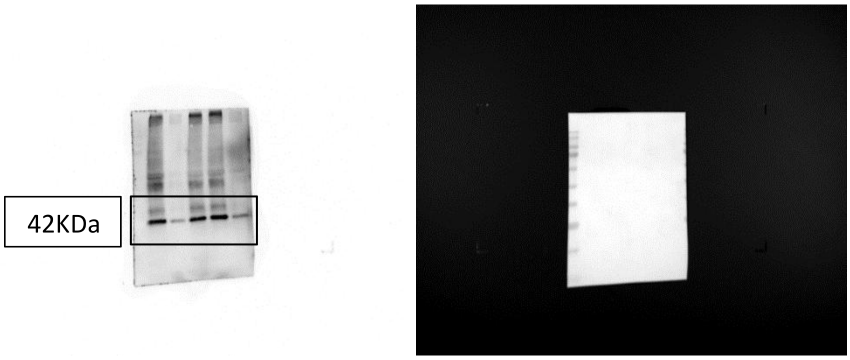

α-SMA

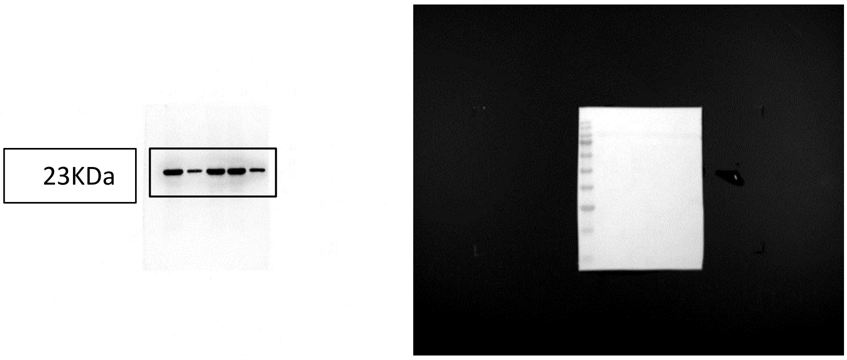

MYH11

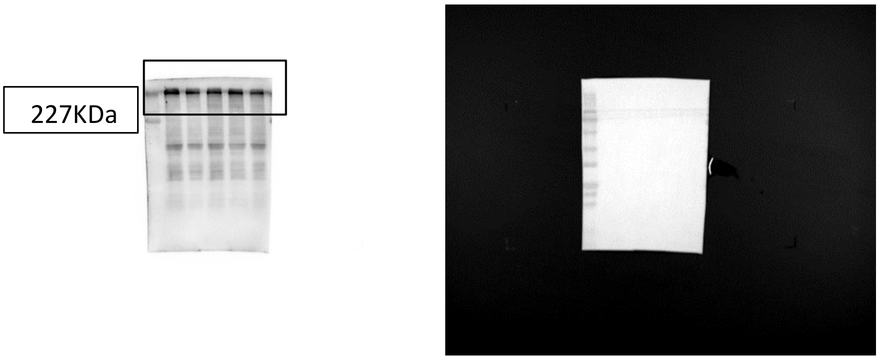

ACTB

42KDa

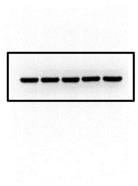

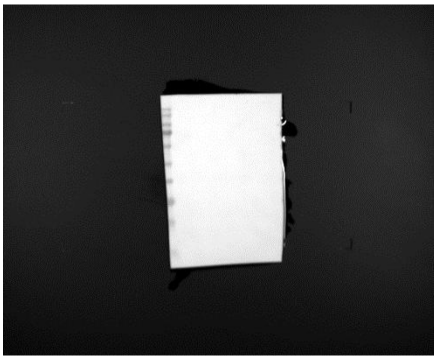

Figure 6

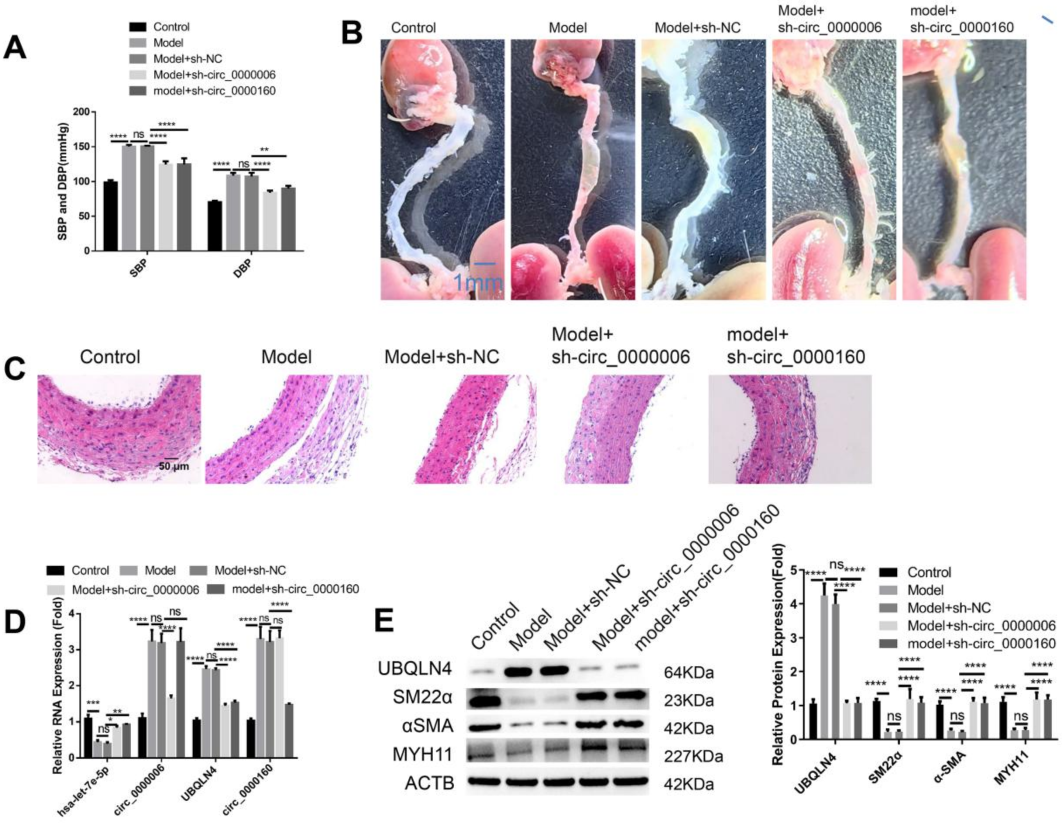

UBQLN4

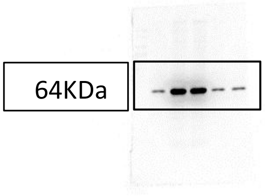

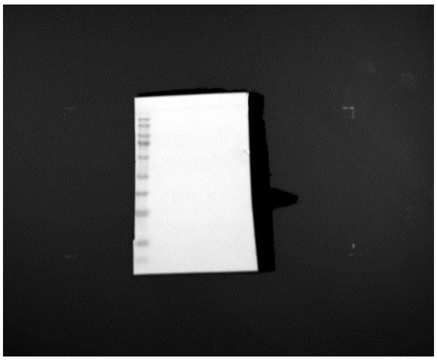

SM22 α

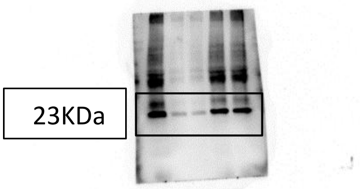

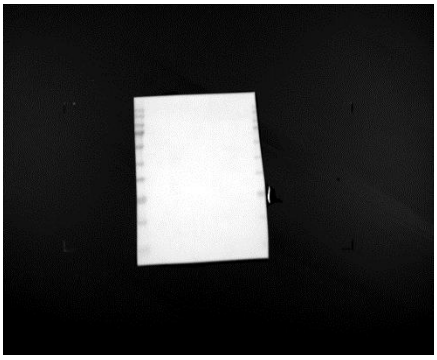

α -SMA

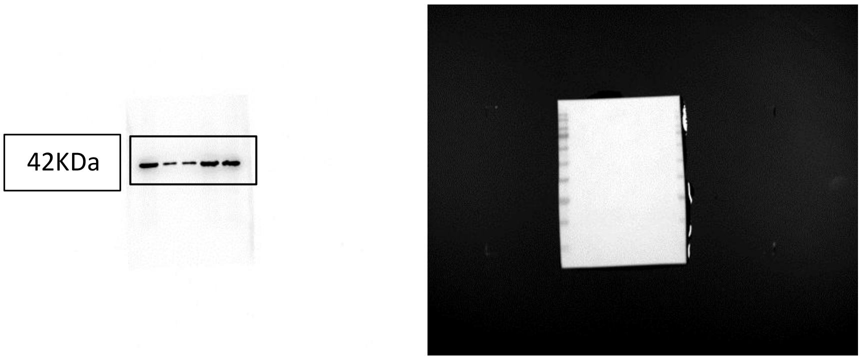

MYH11

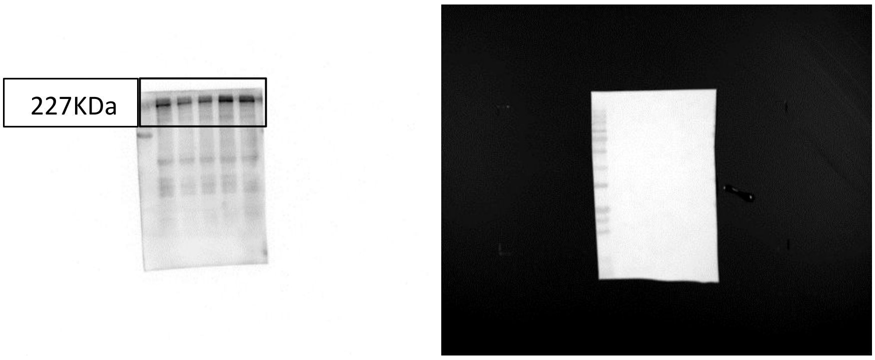

ACTB

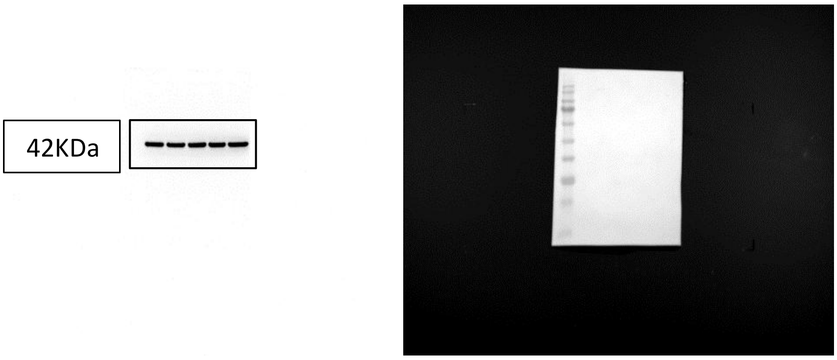

Supplement: S1 Raw images — (PDF) [file pone.0304668.s002.pdf]
